# Perceived stress and its associated factors among pregnant women in Bale zone Hospitals, Southeast Ethiopia: a cross-sectional study

**DOI:** 10.1186/s13104-019-4383-0

**Published:** 2019-06-24

**Authors:** Nigus Alemnew Engidaw, Alemayehu Gonie Mekonnen, Fetene Kassahun Amogne

**Affiliations:** 1 Department of Nursing, College of Health Sciences, Debre Berhan University, Po. Box 445, Debre Berhan, Ethiopia; 2 Department of Midwifery, College of Health Sciences, Debre Berhan University, Po. Box 445, Debre Berhan, Ethiopia

**Keywords:** Perceived stress, Pregnancy, Bale zone hospitals

## Abstract

**Objectives:**

Even though perceived stresses during pregnancy adversely affect the mother and her baby, there is still a scarcity of data from developing countries including Ethiopia. Therefore, this study assessed the prevalence of perceived stress and associated factors among pregnant women in Bale zone hospitals, Southeast Ethiopia. Cross-sectional study was conducted from November 2016 to April 2017. A total of 396 pregnant women were successfully interviewed using structured and pre-tested questionnaires. Perceived stress scale was employed to assess the women’s stress status. A systematic random sampling technique was used. Logistic regression was applied to identify factors associated with perceived stress and statistical significance was considered at p-value < 0.05.

**Results:**

In this study, the prevalence of perceived stress among pregnant women was 11.6% (95% CI 8.30, 14.60). Having 2–5 pregnancies previously (AOR = 9.82; CI 1.08, 89.5) and gestational age less than 12 weeks (AOR = 3.53; CI 1.03, 12.08) were associated with perceived stress among pregnant women. In this study, the prevalence of perceived stress among pregnant women was relatively low. Health care providers should give due attention to the screening of stress in the first trimester to reduce the likelihood of pregnancy-specific stress.

## Introduction

Stress is a complex pattern of a reaction of the human physiology to a demanding situation. It is a process in which we perceive and deal with threats and challenges around us [1]. Even though pregnancy is often considered as an exciting time, it has a stressful journey in a productive woman’s life that needs a significant emotional adjustment [2, 3]. Stress during pregnancy is defined as the imbalance that a pregnant woman feels when she cannot deal with demands and worries [4]. Worldwide, stress is a very common mental health problem among women during their time of pregnancy [5]. Studies reported that the prevalence of stress during pregnancy range from 5.5 to 78% [5–12].

A number of biopsychosocial risk factors contribute to perceived stress during pregnancy. Important factors among them were a past history of depression, domestic violence, stressful life events and interpersonal conflicts [13, 14]. Women who experience high levels of stress are more likely to be from low socioeconomic status [14], less than 20 years of age, being single, have less than grade 11 educations and have no good social support [15]. In a study done Macao, China also showed that women who were separated, divorced or cohabiting were more likely to appraise their lives as stressful [16]. Other researchers have also established that partners conflict during pregnancy leads to pregnancy-related concerns [17, 18] and emotional pain [19]. For example, Poor marital adjustment is known to predict a higher degree of bothers during pregnancy [18]. A Systematic Review among pregnant women illustrates that increased maternal stress was associated with gravidity, gestational age at delivery and monthly family income [1].

Mild level of perceived stress during pregnancy is good for the most favorable development of the fetus, but if it goes beyond it may lead to long term effect on the fetus, and change the development of the fetal nervous system [20]. Stress at the time of pregnancy is also associated with preterm birth and low-birth-weight infants, the risk of gestational hypertension, and undesirable health and behavioral outcomes which lead to infant mortality, cerebral palsy, delays in development, vision and hearing impairments [21, 22]. It has also an effect on the formation of a safe attachment bond with the newborn [23, 24]. Demanding life events before the time of delivery is also linked with the mental health problems in childhood, adolescent and adulthood [25].

Even though perceived stress during pregnancy adversely affects the mother and her baby, there is still a scarcity of data from developing countries including Ethiopia. Therefore, this study assessed the prevalence of perceived stress and associated factors among pregnant women in Bale zone hospitals, Southeast Ethiopia.

## Main text

### Study area and period

The study was conducted from November 2016 to April 2017 in Bale zone hospitals. These hospitals are located in the southeast part of Ethiopia. Robe, the zone city, is located 435 km far from the capital city of Ethiopia; Addis Ababa. The hospitals gave all types of obstetric care including antenatal care and family planning services (*unpublished Bale zone health office report 2016*).

### Study design

A cross-sectional study design was carried out.

### Study population

All pregnant women who attended antenatal care (ANC) service were the source population. Pregnant women who had known sever psychiatric illnesses which might affect the stress status of women were excluded.

### Operational definitions

#### Perceived stress

It was measured with the perceived stress scale (PSS). PSS is a 7-item multiple-choice self-report psychological instrument for measuring the perception of stress. Each answer is scored 0 to 3. PSS is scored by summing across all scale items. The total score ranges 0.0–21.0 [mean = 13.7 (± 6.6)] with higher scores indicating women with more perceived stress symptoms. The cut-off value for the stress limit was set at 15 [26, 27].

### Sample size and sampling procedure

The sample size was determined using a single population proportion formula: a proportion of stress among pregnant women as 37.4% (p = 0.374) taken from a study conducted in Ghana [12], 95% confidence interval (CI) to be 1.96, and margin of error to be 5%. Adding a non-response rate of 10%, the total sample size was 396.

The total sample size was proportionally allocated to each hospital. Pregnant women were selected from the ANC unit systematically. The sampling interval was determined by dividing the number of average monthly ANC services by its sample size. The first woman was selected by lottery method from their order of discharge registration, and every third study participant at the exit of the ANC unit was included in the study.

### Data collection

The structured and pre-tested questionnaire was employed. The questionnaire was prepared first in English from published articles and then translated into Amharic and Afan Oromo (local languages). Data collectors and supervisors were trained for 1 day before the actual data collection. The perceived stress level was measured with the perceived stress scale (PSS). PSS is a 7-item multiple-choice self-report psychological instrument for measuring the perception of stress [26]. Previously it has been used in studies conducted in Ethiopia [27–29].

### Data processing and analysis

Data completeness and inconsistencies were checked. Epi-data version 3.1 was used for data entry and data were exported into SPSS version 22. Logistic regression analyses were applied to identify the association between perceived stress and independent variables. Independent variables that had a significant association in the bivariate analysis were entered into the multivariable analysis. A significant association was declared at a p < 0.05. The results were presented in text and tables with adjusted odds ratio (AOR) and the corresponding 95% confidence interval.

### Results

#### Maternal socio-demographic characteristics

A total of 396 pregnant women were successfully interviewed and the response rate was 93.6%. More than half (52.5%) of women were in the age group of less than 24 years. The mean age of the respondents was 25 (± 5.44) years. Fifty-seven percent (57%) of the women were Muslim and 37.3% were Orthodox Christians. The largest proportions, (94.7%) of the women were married. Concerning the educational level of the respondents, 39.4% of women had completed primary education, whereas 21.2% of women had not attended formal education. Seventy-three percent of pregnant women were living in urban areas. The majority (91.2%) of the pregnant women were living with their husband (Table 1).

**Table 1:** Socio-demographic characteristics of pregnant women in Bale zone, Ethiopia, April 2017 (n = 396)

Variable	Characteristics	Frequency	Percent
Age	≤ 24	208	52.5
	25–34	161	40.7
	≥ 35	27	6.8
Religion	Muslim	227	57.3
	Orthodox	148	37.4
	Protestant	17	4.3
	Others^a^	4	1.0
Marital status	Married	375	94.7
	single	21	5.3
Educational status	No formal education	84	21.2
	Primary education	156	39.4
	Secondary education	87	22.0
	College and above	69	17.4
Occupation	Housewife	234	59.1
	Merchant	49	12.4
	Private employee	31	7.8
	Farmer	14	3.5
	Government employee	52	13.1
	Others^b^	16	4.1
Residence	Urban	289	73.0
	Rural	107	27.0
Living arrangement	With husband	361	91.2
	Alone	35	8.8

^a^Catholic and Waqeefataa

^b^Student

#### Obstetrics characteristics of respondents

In this study, the mean gestational age of participants was 21 (± 8.79) weeks and 45% of women had more than six deliveries (they were multigravida). For 85.6% of participants, their current pregnancy was planned. Around eight percent (7.8%) of the participants reported that they had born low birth weight infant (< 2500 g). Four percent of women reported that a history of molar pregnancy. Approximately two-third (57.8%) of study participants used contraceptives before the current birth (Table 2).

**Table 2:** Obstetrics characteristics of pregnant women in Bale zone, Ethiopia, April 2017

Variable	Characteristics	Frequency	Percent
Gravid	1	139	35.1
	2–5	212	53.5
	≥ 6	45	45
Status of pregnancy	Wanted/planned	339	85.6
	Unwanted/unplanned	57	14.4
Gestational age	≤ 12	86	21.7
	13–24	161	40.7
	≥ 25	25	37.6
Contraceptive use before this pregnancy	Yes	229	57.8
	No	167	42.2
The age of her last baby (in months) (n = 226)	≤ 24	109	48.2
	≥ 25	117	51.8
Pre-pregnancy motion sickness	Yes	116	29.3
	No	280	70.7
History of molar pregnancy (253)	Yes	11	4.3
	No	205	81.0
	Do not know/not sure	37	14.6
Previous pregnancy with low birth weight (258)	Yes	20	7.8
	No	144	55.8
	Do not know/not sure	94	36.4

#### Prevalence and factors associated with perceived stress

Overall, the prevalence of perceived stress among pregnant women was 11.6% (95% CI 8.3, 14.6). In the bivariate analyses, marital status, the age of the respondents, the occupation of the woman, living arrangements, the status of pregnancy, number of pregnancy, gestational age and history of neonatal death during the previous pregnancy were associated with perceived stress. In the multivariable analysis, having 2–5 pregnancies previously and gestational age less than 12 weeks were associated with perceived stress during pregnancy. It was observed that pregnant women who had 2–5 pregnancies were nine times more likely to develop perceived stress than those who had more than 6 pregnancies (AOR = 9.82; CI 1.08, 89.5). In addition, the odds of developing perceived stress was higher among pregnant women who are in early gestational weeks (AOR = 3.53; CI 1.03, 12.08) (Table 3).

**Table 3:** Risk factors associated with stress among pregnant women in Bale zone, April 2017

Variable	Categories	Perceived stress		COR 95% CI	AOR 95% CI
		Yes	No	COR	AOR
Marital status	Married	41	334	1	1
	Single	5	16	2.55 (0.13, 1.12)	1.75 (0.15, 20.07)
Age of the respondents	≤ 24	24	184	3.39 (0.44, 26.14)	2.28 (0.82, 6.37)
	25–34	21	140	3.90 (0.50, 30.27)	1.44 (0.10, 20.28)
	≥ 34	1	26	1	1
Occupation	Housewife	23	212	1	1
	Merchant	5	44	1.07 (0.386, 2.98)	0.71 (0.044, 11.58)
	Private employee	7	43	2.69 (1.04, 6.92)	0.29 (0.02, 14.47)
	Farmer	5	24	1.42 (0.30, 6.92)	2.18 (0.11, 42.2)
	Government employee	6	13	0.78 (0.26, 2.38)	0.64 (0.03, 14.47)
Living arrangements	With husband	38	323	1	1
	Alone	8	27	2.52 (1.07, 5.94)	0.29 (0.016, 5.34)
Status of pregnancy	Wanted/planned	36	303	1	1
	Unwanted/unplanned	10	47	0.56 (0.26, 1.200)	1.12 (0.10, 12.15)
Gravida (in number)	1	9	130	3.05 (0.38, 24.73)	1.08 (0.29, 4.02)
	2–5	36	176	9.00 (1.20, 67.46)	*9.82 (1.08, 89.5)*
	≥ 6	1	44	1	*1*
Gestational age (in weeks)	≤ 12	23	63	6.44 (2.73, 15.12)	*3.53 (1.03, 12.08)*
	13–24	15	146	1.81 (0.75, 4.40)	1.04 (0.34, 3.18)
	≥ 25	8	141	1	1
History neonatal death	Yes	2	30	0.38 (0.09, 1.65)	0.60 (0.11, 3.18)
	No	34	192	1	1

Italic values indicate significance of P-value (P < 0.05)

### Discussion

In this study, the overall prevalence of perceived stress among pregnant women was found to be 11.6% (95% CI 8.3, 14.6). This figure is lower than the study conducted in Ireland (75.6%) [30], Saudi Arabia (33.4%) [7], India (33.3%) [1], Nepal (34.2%) [11] and Ghana (28.6%) [12]. On the other hand, it is higher than the study done in the USA (6%) [5] and Iran (5.5%) [3]. This variation could be due to inadequate sample size, the difference in the geographical area and cultural practices. For example in Ireland, only 74 pregnant women were enrolled in the study [30]. The other possible reason might be, in our study, most of the pregnant women have planned pregnancy and the majority of them were living with their husband. Social support could reduce stress, hiding the effects of stress and protect pregnant women from the harmful effects of stressful situations [8]. Most of the Ethiopian society is more supportive of pregnant women. Accordingly, pregnant women have been excused from social obligations at the time of their pregnancy. Similarly, in most of the Ethiopian women and majority of the society as well, being pregnant has been considered as a blessing especially in the rural, semi-urban and religious communities. This could reduce their level of stress during pregnancy.

In this study, there is no significant association seen between socio-economic status and stress level among pregnant women. This is supported by previous studies [14, 31, 32]. Pregnant women who had 2–5 pregnancies were nine times more likely to develop perceived stress than those who had more than 6 pregnancies. This result is consistent with the previous study [6, 9]. Women who had given birth before could have high levels of pregnancy-related perceived stress especially if their previous pregnancy and delivery experiences were undesirable [33]. In addition, the odds of developing perceived stress is higher among pregnant women who are in early gestational weeks. This contradicts the study in Ghana where perceived stress was higher among pregnant women who are in the third trimester [12]. The possible explanation could be at the early age of pregnant women might fear the possibility of miscarriage/loss of pregnancy and the viability of the fetus.

### Conclusion

In this study, the prevalence of perceived stress among pregnant women was relatively low. Having 2–5 pregnancies previously and gestational age less than 12 weeks were associated with perceived stress during pregnancy. Health care providers should give due attention to the screening of stress in the first trimester in order to reduce the likelihood of pregnancy-specific perceived stress.

## Limitations of the study

This study was conducted in health facilities; hence the findings might not adequately reflect the stresses of the entire pregnant women in the community.

## Data Availability

The datasets generated and/or analyzed during the current study are not publicly available due to some privacy reasons, but part of the row datasets will be available in the recommended publicly available data repository of BMC or from the corresponding author on reasonable request.

## Abbreviations

ANC: antenatal care
AOR: adjusted odds ratio
CI: confidence interval
PSS: perceived stress scale
SPSS: Statistical Package for Social Science
USA: United States of America
